# Silver Nanoparticle-Enhanced Resonance Raman Sensor of Chromium(III) in Seawater Samples

**DOI:** 10.3390/s150510088

**Published:** 2015-04-29

**Authors:** Nguyễn Hoàng Ly, Sang-Woo Joo

**Affiliations:** Department of Chemistry, Soongsil University, Seoul 156-743, Korea; E-Mail: nguyenhoangly2007@gmail.com

**Keywords:** Cr(III), EDTA, Raman spectroscopy, silver nanoparticles, seawater

## Abstract

Tris(hydroxymethyl)aminomethane ethylenediaminetetraacetic acid (Tris-EDTA), upon binding Cr(III) in aqueous solutions at pH 8.0 on silver nanoparticles (AgNPs), was found to provide a sensitive and selective Raman marker band at ~563 cm^−1^, which can be ascribed to the metal-N band. UV-Vis absorption spectra also supported the aggregation and structural change of EDTA upon binding Cr(III). Only for Cr(III) concentrations above 500 nM, the band at ~563 cm^−1^ become strongly intensified in the surface-enhanced Raman scattering spectra. This band, due to the metal-EDTA complex, was not observed in the case of 50 μM of K^+^, Cd^2+^, Mg^2+^, Ca^2+^, Mn^2+^, Co^2+^, Na^+^, Cu^2+^, NH_4_^+^, Hg^2+^, Ni^2+^, Fe^3+^, Pb^2+^, Fe^2+^, and Zn^2+^ ions. Seawater samples containing K, Mg, Ca, and Na ion concentrations higher than 8 mM also showed the characteristic Raman band at ~563 cm^−1^ above 500 nM, validating our method. Our approach may be useful in detecting real water samples by means of AgNPs and Raman spectroscopy.

## 1. Introduction

Recent developments in methodologies for the detection and quantitation of heavy metal ions in aqueous solutions, are now receiving much attention [1]. Nanoparticles have been introduced for sensing heavy metal ions in solutions [2]. In this study, a strong metal ion chelator of ethylenediaminetetraacetic acid (EDTA) was found to make the chelation/aggregation process reversible. Raman spectroscopy is currently the method of choice for detection of trace analysis and surface characterization [3,4]. Surface-enhanced Raman spectroscopy (SERS) has recently been used to detect trace amounts of several hazardous substances [5,6]. Ultra-sensitive detection of ions is feasible through the combination of aggregation-dependent noble metal nanoparticles (NPs) and probe material in the field of nanobioanalysis [7].

Trivalent chromium is used as a dietary supplement and a therapeutic agent to increase the sensitivity of insulin [8,9]. The quantification of Cr(III) in water has usually required conventional approaches of various analytical techniques [10,11,12,13]. Metal nanostructures have been recently introduced to detect chromium ions with a combination of several linker units including Tween 20 [14], glutathione [15], 11-mercaptoundecanoic acid [16], 5,5'-dithio-*bis*-(2-nitrobenzoic acid) [17,18], citric acid [19], and a series of heterotripodal receptors [20]. SERS detection of Cr^3+^ has been reported using AgNPs and lateral flow immunoassays [21]. The monothiocyanate complexes of Cr^3+^ were also investigated on the silver electrode by SERS [22]. 

EDTA has been used to detect various foreign ions [23,24]. EDTA is known to show a strong Raman signal at 40–500 cm^−1^ due to the metal-N stretching bands, with a complex of several ionic species [25,26,27]. In the present work, we have introduced EDTA, as a method of confirming dissimilar adsorption behaviors depending on the concentrations of Cr(III) in the complex that yields a prominent spectral intensity at ~563 cm^−1^ on silver nanoparticles (AgNPs) at concentrations of Cr(III) higher than 500 nM. Infrared spectroscopy also supported a structural change of EDTA upon binding Cr(III). UV-Vis absorption spectra suggest that there should be a resonance electron transition for the EDTA-Cr(III) complex and the plasmonic shifts of AgNPs at 600~800 nm. 

We report a Cr(III) detection method with a complex of EDTA and subsequent adsorption on AgNPs by means of resonance Raman spectroscopy using a He-Ne laser excitation at 633 nm. Considering that the Environmental Protection Agency (EPA) regulation [1] for levels in drinking water of the Cr(III) ion is around 1.9 × 10^−6^ M, our method may be useful in detecting harmful wastewater by means of AgNPs and SERS. The EDTA-Cr(III) complex could yield different SERS intensities on AgNPs. EDTA is found to be sensitive and selective in binding Cr(III) in aqueous AgNP solutions.

## 2. Experimental Section 

### 2.1. Chemicals

Cr(III) and the other ionic substances of NaCl, KNO_3_, Mg(NO_3_)_2_, Ca(NO_3_)_2_, Cr(NO_3_)_3_, Mn(NO_3_)_2_, FeC_2_O_4_, Fe(NO_3_)_3_, Co(NO_3_)_2_, Ni(NO_3_)_2_, Cu(NO_3_)_2_, Zn(NO_3_)_2_, NH_4_NO_3_, Cd(NO_3_)_2_, Hg(NO_3_)_2_, Pb(NO_3_)_2_, and K_2_Cr_2_O_7_ (or Na_2_CrO_4_) along with silver nitrate, sodium citrate, Tris-EDTA (pH 8.0), and EDTA acetic acid were purchased from Sigma Aldrich (St. Louis, MO, USA). The buffer solution of pH 4.0 was purchased from Thermo Scientific (Waltham, MA, USA). The seawater samples were obtained from the Yellow Sea (Tae-an, Chungcheongnam-do, Korea). The citrate-stabilized silver colloidal substrates were synthesized by following the recipes in the literature [28].

### 2.2. Equipment and Characterization Methods

In order to observe the resonance processes and aggregation properties of AgNPs, UV-Vis absorption spectra were obtained using a Mecasys UV-3220PC spectrophotometer (Daejeon, Korea). The transmission electron microscope images of AgNPs were obtained using JEM-3010 and JEM-1010 microscopes from JEOL (Peabody, MA, USA). For Fourier transform infrared spectroscopy (FT-IR) data, a drop of the EDTA-Cr(III) solution sample was transferred onto a MIRacle ZnSe attenuated-total reflection accessory from Pike Technologies (Madison, WI, USA). The infrared spectra were obtained using a FT-IR 6700 spectrometer equipped with a narrow band HgCdTe detector at the cut-off of 800 cm^−1^ from Thermo Nicolet (Waltham, MA, USA). Raman spectra at the irradiation of 632.8 nm were obtained using the previous method [29]. The resonance Raman spectrum at 532 nm in comparison with off-resonance near-infrared Raman at 785 nm was obtained using a LabRam Aramis spectrometer from Horiba Jovin Yvon (Palaiseau, France). X-ray photoelectron (XPS) spectra were obtained using a Sigma Probe instrument from Thermo VG using an Al-Kα monochromatic source. 

### 2.3. Sample Preparation 

For the SERS experiment, 10 µL Cr^3+^ (1 mM, in either distilled water or seawater) and 50 µL EDTA (Tris-EDTA buffer solution, pH 8.0) were mixed and stirred for over 60 min at room temperature. Subsequently, 140 µL of AgNPs were added to the mixture and the SERS spectra were observed. Seawater obtained from the Yellow Sea in Korea was checked by inductively coupled plasma optical emissions spectrometry (ICP-OES) with an ICAP-7400 analyzer from Thermo Scientific (Waltham, MA, USA) and a CETAC M-7500 mercury analyzer from Parma Company (Omaha, NE, USA). AgNPs-EDTA exhibited a selective turn-on Raman intensity response to Cr^3+^ in seawater (50 µM). Response behaviors were observed for AgNPs-EDTA in the presence of various environmentally relevant metal ions. The Raman spectral features of AgNPs-EDTA-M^n+^ were unchanged in the presence of K^+^, Cd^2+^, Mg^2+^, Ca^2+^, Mn^2+^, Co^2+^, Na^+^, Cu^2+^, NH_4_^+^, Hg^2+^, Ni^2+^, Fe^3+^, Pb^2+^, Fe^2+^, and Zn^2+^ ions (all at concentrations of 50 µM). Of all the tested metal ions, only the Cr^3+^ ion increased the Raman intensity in the presence of AgNPs-EDTA. For the XPS experiment, 1.0 mL of this mixture was put into a 1.5 mL centrifugal tube, the AgNPs-EDTA-Cr^3+^ was collected by centrifugation at 10,000 rpm for 10 min at 4 °C, and the supernatant ones were carefully removed up to a residual volume of 20 µL. The 20 µL of AgNPs-EDTA-Cr^3+^ was then dropped onto a cover glass (size 5 mm × 5 mm) from Deckgläser (Sondheim/Rhön, Germany), and dried at 75 °C overnight, and the XPS spectra were then observed.

## 3. Results and Discussion

**Figure 1:**
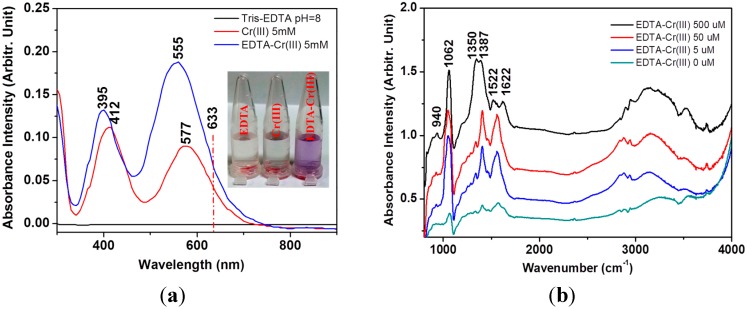
(**a**) UV-Vis spectra of ethylenediaminetetraacetic acid (EDTA), Cr(III), and EDTA-Cr(III). The inset shows the photo images. The dotted line indicates 633 nm; (**b**) Fourier transform infrared (FT-IR) spectra of EDTA-Cr(III) at 800–4000 cm^−1^.

Figure 1a shows the photographic images and UV-Vis absorption spectra of EDTA-Cr(III). Upon binding Cr(III), the color changed to purple to increase the absorbance values at 500–700 nm, including 633 nm of the excitation wavelength of the Raman experiments. Infrared spectra also suggested the structural change, as indicated in Figure 1b. The UV-Vis spectrum of EDTA appeared to be similar to the previous report [30]. The bands at 1622, 1522, and 1387 cm^−^^1^ can be ascribed to the ν(C=O), ν_as_(COO^−^), and ν_s_(COO^−^) bands, respectively, [31]. The spectral changes suggest that more symmetric vibrations of the COO^−^ band became stronger instead of the anti-symmetric vibration.

**Figure 2:**
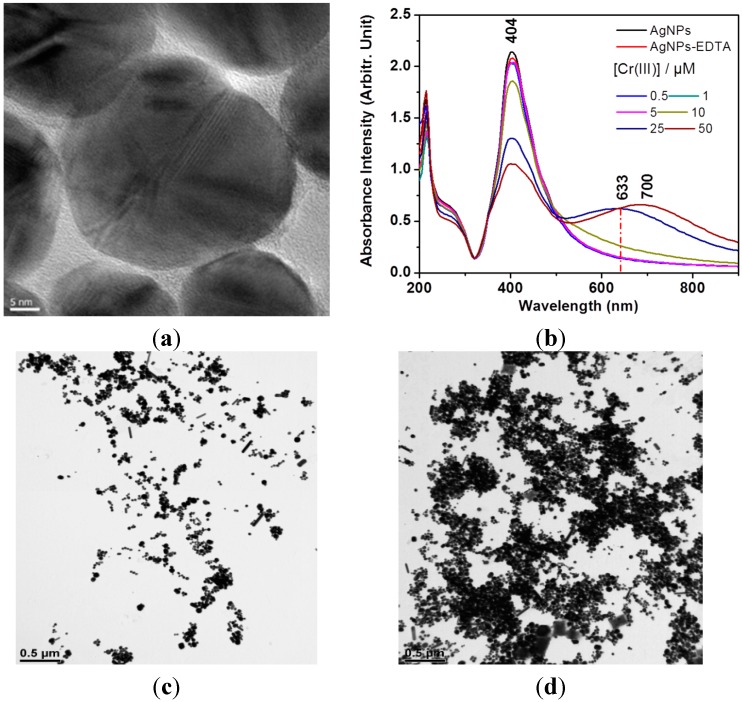
(**a**) TEM image of silver nanoparticles (AgNPs). The scale bar indicates 5 nm; (**b**) UV-Vis absorption of AgNPs upon binding of EDTA with Cr(III). TEM images of aggregated AgNPs with EDTA at [Cr^3+^] of (**c**) 0 and (**d**) 50 μM. The scale bars indicate 0.5 μM.

Figure 2 shows a TEM image of prepared AgNPs. Upon the increase of Cr(III) in the complex, the UV-Vis spectra of AgNPs became red-shifted toward 600–800 nm to suggest the aggregation of AgNPs. These results indicate that EDTA with higher concentrations of Cr(III) could induce the plasmonic shift of the AgNPs. It has to be admitted that, it was not easy to compare the aggregation by the higher concentration of Cr^3+^ due to the dryness of the sample on the grid. The representative images depending on the concentrations of Cr^3+^ are compared in Figure 2c,d.

**Figure 3:**
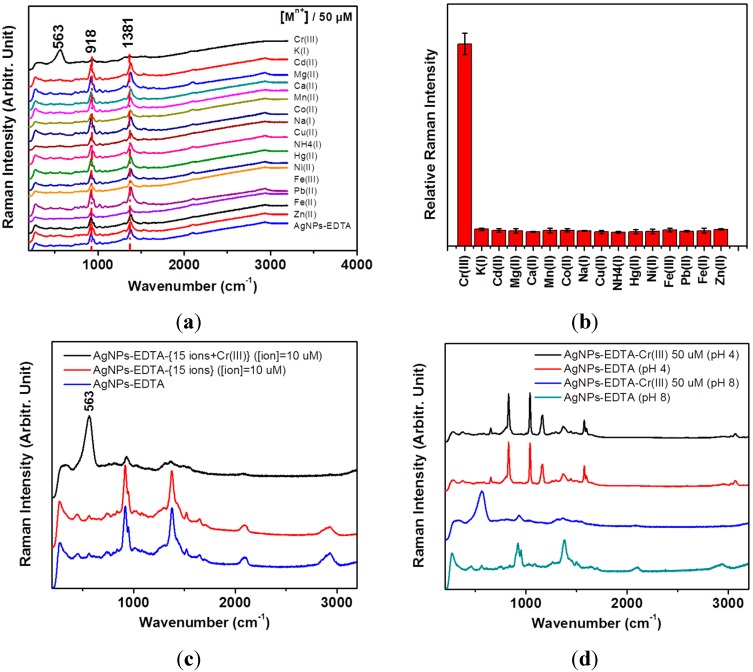
(**a**) Surface-enhanced Raman spectroscopy (SERS) spectra of detection of Cr(III) using Tris(hydroxymethyl)aminomethane (Tris)-EDTA on AgNPs. Selective tests were performed for 50 μM of Cr^3+^, K^+^, Cd^2+^, Mg^2+^, Ca^2+^, Mn^2+^, Co^2+^, Na^+^, Cu^2+^, NH_4_^+^, Hg^2+^, Ni^2+^, Fe^3+^, Pb^2+^, Fe^2+^, and Zn^2+^ ions; (**b**) Stick diagram of the intensities at ~563 cm^−1^ with respect to those at ~918 cm^−1^ *versus* 16 ionic species; (**c**) SERS spectra of the mixture of the 15 ions to test the competitive reactions; (**d**) Comparative SERS spectra of EDTA and EDTA-Cr^3+^ on AgNPs at pH 4.0 and pH 8.0.

The increase of the absorbance at 633 nm should be more suitable for the surface-enhanced resonance Raman scattering (SERRS) experiments as performed in Figure 3. We attempted to observe the surface-enhanced resonance Raman spectra via 532 nm instead of 632.8 nm. We could clearly see the vibrational band at ~563 cm^−1^ at the excitation of 532 nm, whereas this peak was not observed at 785 nm. This result indicates that the strong Raman band at ~563 cm^−1^ may be due to a resonance Raman effect via the absorption band at 500–600 nm.

Only the Cr^3+^ ion exhibited a unique intense peak at ~563 cm^−1^ of the metal-N stretching band [25,26,27], along with the decrease of the two bands at 918 and 1381 cm^−1^. We also found that the mixture of the other 15 ions of K^+^, Cd^2+^, Mg^2+^, Ca^2+^, Mn^2+^, Co^2+^, Na^+^, Cu^2+^, NH_4_^+^, Hg^2+^, Ni^2+^, Fe^3+^, Pb^2+^, Fe^2+^, and Zn^2+^ with the individual concentration of 10 μM for each ion did not produce such a strong marker band as the case of Cr^3+^. When the 15 ions were mixed with Cr^3+^, the band at 563 cm^−1^ became more intensified as shown in Figure 3c. This result indicated that the other ions may not substantially interfere with the Cr^3+^ ion detection by competitive reactions at the high ion concentrations of 10 μM. We tested the pH-dependent binding behaviors for Cr^3+^ at pH 4.0 using EDTA acetic acid and the buffer solution at pH 4.0. We found that the Raman spectrum at pH 4.0 became quite different from that at pH 8.0. Moreover, the Raman spectrum at pH 4.0 did not produce such a peak of 563 cm^−^^1^ at pH 8.0, as compared in Figure 3d. This result indicated that the alkaline pH value of the AgNPs should be essential to producing the EDTA-Cr^3+^-induced spectral changes.

**Figure 4:**
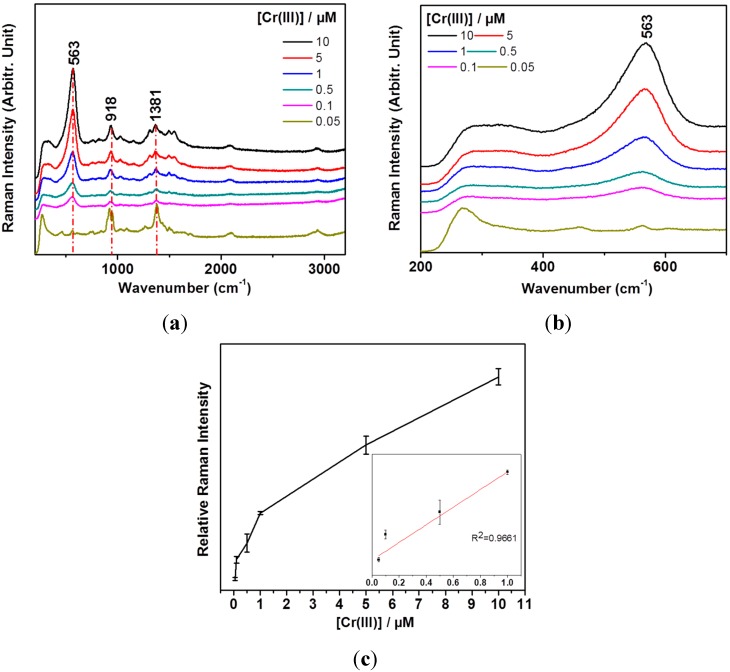
(**a**) Concentration-dependent SERS spectra using AgNPs and Tris-EDTA in the range of 0.005–10 μM of Cr(III); (**b**) A magnified view of the vibrational bands between 200 and 750 cm^−1^ in the Cr(III) concentration-dependent SERS spectra; (**c**) A calibration curve of the vibrational band intensities at ~563 cm^−1^ with respect to those at ~918 cm^−1^. The inset shows a linear fitting result for the 0.05 and 1.0 μM regions.

The two bands at 918 and 1381 cm^−1^, can be ascribed to the ν(C-C) and ν_s_(COO^−^) bands, respectively, according to the literature [29]. The stronger enhancements of the νs(COO^−^) bands in the SERS spectrum in comparison with the FT-IR spectra may be due to the different selection rules. The two strong bands could be found at low concentrations of Cr(III) below either 500 nM, or 10 μM of K^+^, Cd^2+^, Mg^2+^, Ca^2+^, Mn^2+^, Co^2+^, Na^+^, Cu^2+^, NH_4_^+^, Hg^2+^, Ni^2+^, Fe^3+^, Pb^2+^, Fe^2+^, and Zn^2+^ ions. Only with the Cr(III) concentration above 500 nM did the band become strongly intensified at 563 cm^−1^. This may be due to unique Raman enhancements of EDTA-Cr(III) for metal-N stretching band on AgNPs. Since EDTA compounds are known to combine various ions, it is likely that the EDTA-ion complex may have a different interaction on AgNPs only in the case of Cr^3+^. According to the X-ray photoelectron spectroscopy (XPS) data, the atomic percentages of the EDTA complexes of Cr and K on AgNPs were measured to be 1.41% and 0.24%, respectively. The relative percentages of Cr and K with respect to Ag were also found to be 3.93% and 0.77%, respectively. For the case of the Fe^3+^ ion, we could not observe any iron species in the whole spectral region between 1000 and 0 eV. Our XPS data suggest that the Cr^3+^ ion may strongly adsorb on Ag surfaces in comparison with the other ions. These results suggest that the EDTA-Cr^3+^ complex may bind more strongly on AgNPs to lead to strong concentration-dependent SERS spectra. It can be interpreted that EDTA may show the carboxylate bands at lower concentrations of Cr(III) and the other ionic cases. At higher concentrations of Cr(III), the marker band of the Cr(III)-EDTA complex could be intensified in the SERRS spectra. Figure 4a shows concentration-dependent SERS spectra of detection of Cr(III) using AgNPs and tris-EDTA in the range of 0.005–10 μM. We found that the band at 563 cm^−1^ became stronger above 500 nM in distilled water. The SERRS bands from 200 to 300 cm^−1^, which can be ascribed to Ag-N and Ag-COO, also showed different binding of the EDTA-Cr(III) complex on AgNPs.

**Figure 5:**
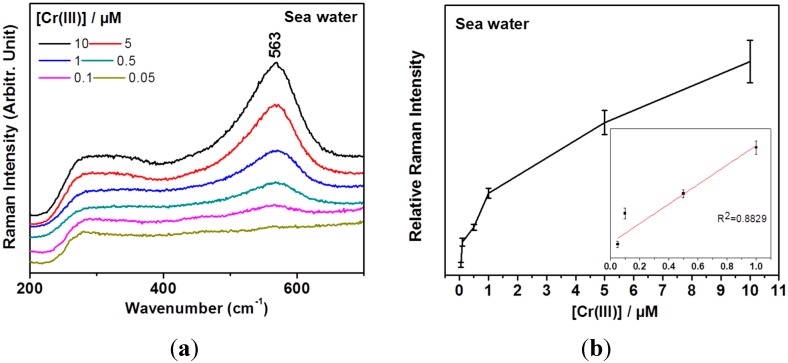
(**a**) Test of real seawater sample for the Cr(III) detection; (**b**) A calibration curve of the vibrational band intensities. Three independent measurements were performed to yield the standard deviations as marked in the error bars. The inset shows a linear fitting result for the 0.05 and 1.0 μM regions.

Based on these data, we attempted to detect Cr(III) by mixing seawater as proof of application. Table 1 shows the elemental compositions of the used seawater where K, Mg, Ca, and Na ion concentrations were ~13.3 mM, ~43.6 mM, ~8.61 mM, and ~435 mM, respectively. We could clearly observe the marker band of 563 cm^−1^ to quantify Cr(III) concentrations as shown in Figure 5a. Figure 5b shows a calibration curve of the vibrational band intensities at ~563 cm^−1^ depending on Cr(III) concentrations in the seawater sample. The detection limit is estimated to be as low as 500 nM.

**Table 1:** The element compositions of the tested water samples. (Unit in ppm (mg/L).)

Sample	Compositions													
	K	Cd	Mg	Ca	Mn	Co	Na	Cu	Hg	Ni	Fe	Pb	Zn	Cr
Distilled	ND *	ND	ND	0.03	ND	ND	1.05	ND	ND	ND	ND	ND	ND	ND
Seawater	521.78	ND	1,059.81	344.90	0.03	ND	10,001.92	0.02	ND	ND	ND	ND	0.02	ND

* ND stands for “Not Detected”. Hg contents were determined by a mercury analyzer, whereas the rest of ions were tested by ICP-OES.

**Figure 6:**
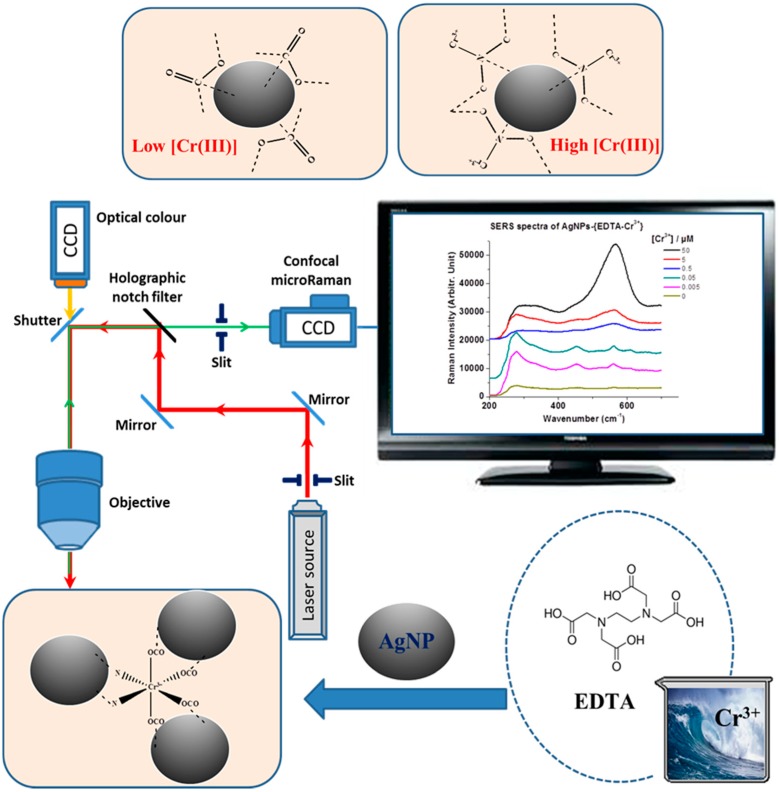
Schematic diagram of detection of Cr(III) using AgNPs. The carboxylic acid group of EDTA is expected to bind on AgNPs at low concentrations of Cr(III). As the Cr(III) increased, the intensified Cr(III)-N mode could be monitored by confocal SERRS.

One of the possibilities of the characteristic Raman band enhancement mechanism is inducing a charge-transfer (CT) transition due to the combined molecule-metal interaction [32]. The energy levels of EDTA-Cr^3+^ and the Fermi level of Ag are resonating for a CT transition, while it is likely that the other EDTA-ion complexes seem not to have an efficient CT transition with Ag in this energy state.

Figure 6 shows a schematic diagram to detect the Cr(III) by means of confocal Raman spectroscopy. EDTA is first introduced to selectively bind Cr(III). On AgNPs, the SERRS spectra in the presence of Cr(III) above 500 nM provide a method to discriminate and quantify Cr(III), despite the interference of K, Mg, Ca, and Na ions in seawater samples. The adsorption geometries for low and high concentrations of Cr(III) were estimated to be different on the basis of Raman spectral features. The strong band at ~563 cm^−1^ suggested a metal-N coordination at high concentrations of Cr(III). Our approach combined the use of SERRS to check the selectivity and sensitivity of the detection of Cr(III) in real seawater solutions. 

## 4. Conclusions

We report a new approach for the submicromolar detection of Cr(III) ions using Tris-EDTA buffer and AgNPs. A quantitative trend down to the micromolar range was observed in the analysis of the spectroscopic changes depending on the Cr(III) concentrations. Tris-EDTA is sensitive and selective in binding chromium (III) in the presence of AgNPs. A detection limit as low as 500 nM of Cr(III) could be achieved on the basis of SERS measurements. Seawater samples containing different chromium contents were also tested by means of Raman spectral change for a practical application of our methods. The EDTA-Cr(III) complex produced a prominent Raman peak at ~563 cm^−1^ on AgNPs at concentrations of Cr(III) higher than 500 nM. Infrared spectroscopy also indicated a structural change of EDTA upon binding Cr(III), whereas UV-Vis absorption spectra suggested that there would be an electron transition at ~600 nm for EDTA-Cr(III) complex and aggregation of AgNPs as the Cr(III) concentration increased. Considering that EPA regulatory limit of the Cr(III) ion for drinking water is around 1.9 × 10^−6^ M, our method may be useful in detecting harmful wastewater by means of AgNPs and SERS. The EDTA-Cr(III) complex could yield different SERRS intensities on AgNPs. Raman spectroscopy is found to be sensitive and selective in binding Cr(III) with EDTA enhanced by AgNPs.
